# Supplementary material for: Nanoimprinted Plasmonic Crystals for Cost-Effective SERS Identification of Methylated DNAs
Source: Sensors (Basel). 2024 Jul 16;24(14):4599. doi: 10.3390/s24144599 (PMC11280517; doi:10.3390/s24144599)
Supplement: Supplementary file 1 [file sensors-24-04599-s001.zip › sensors-3096535-supplementary.pdf]

Nanoimprinted Plasmonic Crystals for Cost-Effective SERS Identification of Methylated DNAs

Daiki Kawasaki[1], Ryosuke Nishituji[2], Tatsuro Endo[3], Hideaki Hisamoto[3,*]

[1] Metamaterials Laboratory, RIKEN Cluster for Pioneering Research, 2-1 Hirosawa, Wako, Saitama 351-0198, Japan

[2] Department of Information Networking, Graduate School of Information Science and Technology, Osaka University, 2-8 Yamadaoka, Suita, Osaka 565-0871, Japan

[3] Department of Applied Chemistry, Graduate School of Engineering, Osaka Metropolitan University, Sakai, Osaka 599-8531, Japan

* Correspondence: t_endo@omu.ac.jp

Contents

Table S1. DNA sequences.

Figure S1. Reflection spectroscopic setup.

Figure S2. Numerically calculated refection spectrum of PNHs and PNDs.

Figure S3. Electric field enhancement by PNHs and PNDs.

Table S1. DNA sequences

	Sequence (5'-3')
**Probe**	HS-GAT TAC ACA GCT GCT TCG CTC TCC GCT TCC CGA CCC GCA C
**Unmethylated target**	GTG CGG GTC GGG AAG CGG AGA GCG AAG CAG CTG TGT AAT C
**Methylated target**	GTC mCGG GTmC GGG AAG CGG AGA GmCG AAG CAG CTG TGT AAT C

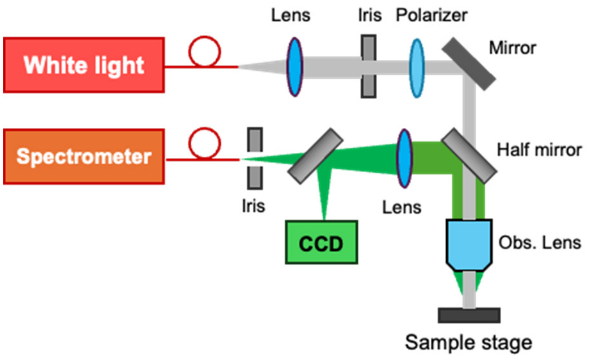

Figure S1. Reflection spectroscopic setup.

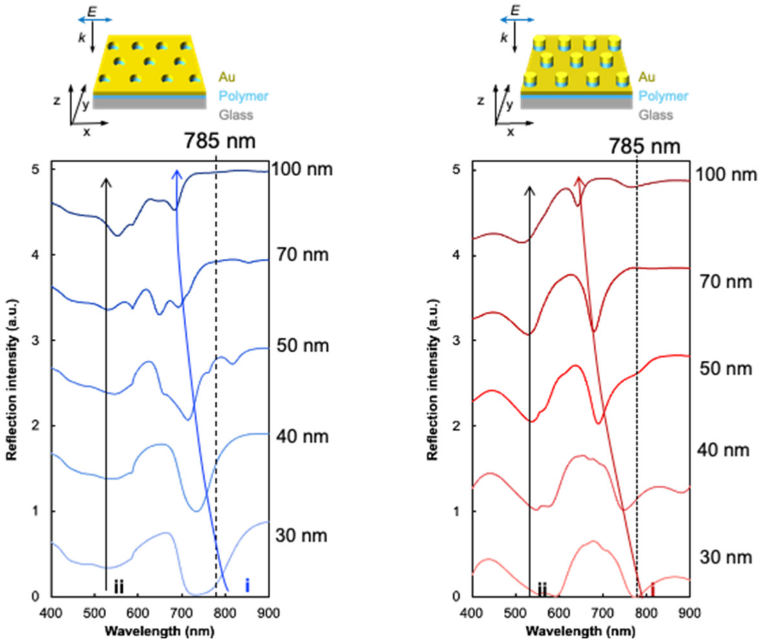

Figure S2. Numerically calculated refection spectrum of PNHs and PNDs.

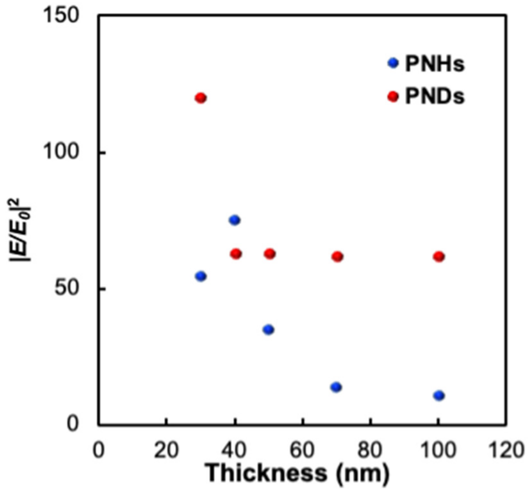

Figure S3. Electric field enhancement by PNHs and PNDs.
